# SbI_3_·3S_8_: A Novel Promising Inorganic Adducts Crystal for Second Harmonic Generation

**DOI:** 10.3390/ma16031105

**Published:** 2023-01-27

**Authors:** Tushar Kanti Das, Marcin Jesionek, Mirosława Kępińska, Marian Nowak, Michalina Kotyczka-Morańska, Maciej Zubko, Jarosław Młyńczak, Krzysztof Kopczyński

**Affiliations:** 1 Institute of Physics—Center for Science and Education, Silesian University of Technology, Krasińskiego 8, 40-019 Katowice, Poland; 2 Institute for Chemical Processing of Coal, Zamkowa 1, 41-803 Zabrze, Poland; 3 Institute of Materials Engineering, Faculty of Science and Technology, University of Silesia, 75 Pułku Piechoty 1a, 41-500 Chorzów, Poland; 4 Department of Physics, Faculty of Science, University of Hradec Králové, Rokitanského 62, 500 03 Hradec Králové, Czech Republic; 5 Institute of Optoelectronics, Military University of Technology, Gen. Sylwestra Kaliskiego 2, 00-908 Warsaw, Poland

**Keywords:** nonlinear optical, SbI_3_·3S_8_ crystal, deep-ultraviolet, middle- and far-infrared, second harmonic generation

## Abstract

In the past twenty years, the basic investigation of innovative Non-Linear Optical (NLO) crystals has received significant attention, which has built the crucial heritage for the use of NLO materials. Fundamental research is essential given the scarcity of materials for NLO compounds, especially in the deep ultraviolet (DUV) and middle- and far-infrared (MFIR) regions. In the present work, we synthesized high-quality MFIR SbI_3_·3S_8_ NLO crystals having a length in the range of 1–5 mm through rapid facile liquid phase ultrasonic reaction followed by the assistance of instantaneous natural evaporation phenomenon of the solvent at room temperature. X-ray diffraction (XRD) results ratify the hexagonal R3m structure of SbI_3_·3S_8_ crystal, and energy-dispersive X-ray spectroscopy (EDX) demonstrates that the elemental composition of SbI_3_·3S_8_ crystal is similar to that of its theoretical composition. The direct and indirect forbidden energy gaps of SbI_3_·3S_8_ were measured from the optical transmittance spectra and they were shown to be 2.893 eV and 1.986 eV, respectively. The green sparkling signal has been observed from the crystal during the second harmonic generation (SHG) experiment. Therefore, as inorganic adducts are often explored as NLO crystals, this work on the MFIR SbI_3_·3S_8_ NLO crystal can bring about additional investigations on this hot topic in the near future.

## 1. Introduction

In recent years, the high demand for laser technology has drawn much more concentration on Non-Linear Optical (NLO) materials [1,2,3,4]. This application requires a large value of second-order non-linear (i.e creation of light at a second-harmonic frequency) vulnerability and an extended range of optical transparency [5,6,7]. The optical nonlinear compound with these characteristic features, which forms a noncentrosymmetric crystal structure in space, is of distinctive attraction [8,9,10]. To procure such an NLO crystal structure, the logical constructions of their elemental compositions are crucial. It is very well known that the coefficient of NLO is inversely proportional to the structural unit of the acentric crystallographic form [11,12]. Researchers are already trying to develop potential NLO crystals by combining two or more numbers of acentric structural units. The reason behind this combination of different structural units used to make NLO crystals is that different structural units exhibit various properties, and their combination provides such properties to NLO crystals [13,14,15].

Still, the developed deep ultraviolet (DUV) and middle- and far-infrared (MFIR) NLO crystals are inadequate to encounter the market requirements. There are many standard DUV NLO crystals available, such as KH_2_PO_4_, KBe_2_BO_3_F_2_, KTiOPO_4_, BPO_4_, β-BaB_2_O_4_, and KLa(PO_3_)_4_, which can satisfy basic necessity of the laser industry [6,16,17,18,19]. On the other hand, though the materials such as KBe_2_BO_3_F_2_, ZnGeP_2_, AgGaS_2_ (AGS), and AgGaSe_2_ (AGSe) have been used as DUV and MFIR NLO crystals, their ingrained obstructions harshly constrict their wide range of applications [20,21]. Among them, the most outstanding NLO crystal for MFIR is AGS and AGSe. However, they still have inherent defects, which make them a potential MFIR NLO material. Hence, it is essential to explore new promising MFIR NLO crystals with much more balance achieved.

**Figure 1:**
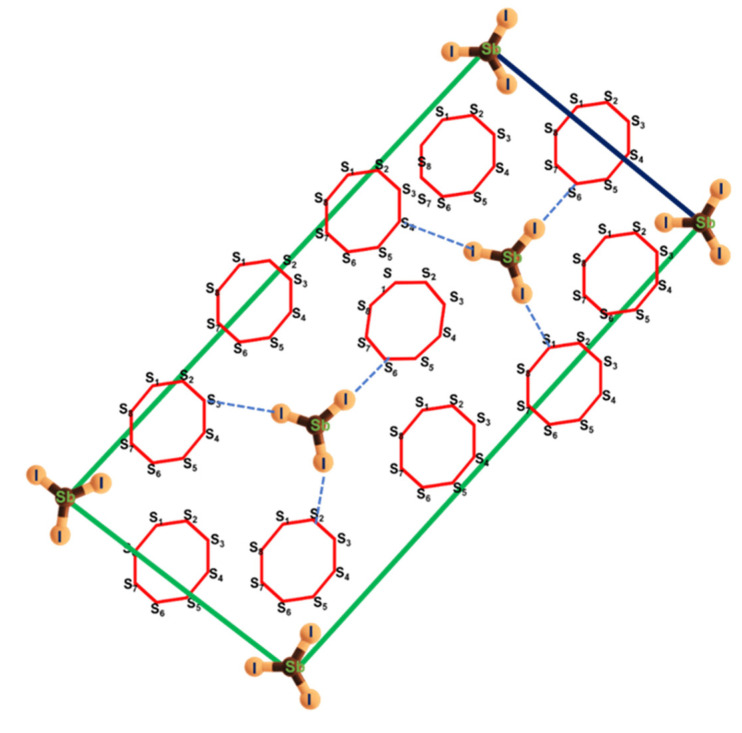
Schematic diagram of SbI_3_·3S_8_ crystal structure.

The MFIR NLO crystals, which are prepared by chalcogenides, halides, and iodates, have a basic structural unit consisting of anions or anionic groups according to Chen’s anionic group hypothesis [22]. This type of crystal is basically formed either by conventional ionic or covalent bonds. The reason behind the fabrication of chalcogenides- and halides-based MFIR NLO crystals is that chalcogenides have a better NLO efficiency, but, in comparison with halides, they have small laser-induced damage thresholds (LIDT) [23]. Therefore, to overcome their deficiency, chalcogenides have been combined with halides in a specific ratio to obtain highly efficient MFIR NLO crystals [24]. Recently, a non-centrosymmetric adduct of MFIR NLO crystal known as antimony triiodide–octasulfur (SbI_3_·3S_8_) was explored, and it was found to display strong second-order non-linear properties [25], and the schematic non-centrosymmetric structure of the inorganic adduct SbI_3_·3S_8_ crystal is presented in Figure 1. SbI_3_·(S_8_)_3_ crystallizes in the trigonal noncentrosymmetric space group R3m. As presented in Figure 1, in one asymmetric structure unit, there are five S atoms, three I atoms, and one Sb atom. Two asymmetric structure units, trigonal-pyramidal SbI_3_ and chairlike S_8_, are bonded through van der Waals force interaction.

The nature of the essential MFIR NLO SbI_3_·3S_8_ crystals has been explored by several research groups. For example, Fernando et al. synthesized SbI_3_·3S_8_ crystal by the slow evaporation of the carbon disulphide from the constituent, but the quality of the crystals was not good [26]. Bjorvatten et al. also prepared the crystal through the same procedure and investigated its structure through the X-ray diffraction method only [27]. Currently, the crystals have also been fabricated by several other research groups through vapor phase reactions of antimony triiodide (SbI_3_) and sulfur (S), and the reactions were passed through a much more complicated and time-consuming process [25,28,29]. Based on the above background, we feel that it is essential to synthesize such MFIR NLO crystals through facile and rapid methods and explore their properties through practical SHG experiments.

In the present study, we demonstrate the synthesis of MFIR SbI_3_·3S_8_ crystals through a facile and rapid sonochemical process. We report, for the first time, the synthesis of MFIR SbI_3_·3S_8_ crystal from elemental antimony (Sb) sulfur (S), and iodine (I) by conducting sonochemical reaction in dichloromethane (CH_2_Cl_2_) as a solvent, and the natural phenomenon of rapid solvent evaporation helped in the formation of the crystal without the use of any other energy sources. The method is very simple and fast compared to others for producing the best quality SbI_3_·3S_8_ crystal with a high yield. The quality of the crystals was investigated by simple optical microscopy. The grown crystal was identified by X-ray diffraction studies and using the electron microscopic technique. The elemental compositions of the synthesized crystal and their distribution were diagnosed by an energy-dispersive X-ray spectroscopy (EDS) study. Direct and indirect forbidden energy gaps of the grown SbI_3_·3S_8_ crystals were measured by optical transmittance spectra. The second harmonic generation of light (SHG) in the grown crystals was also observed. We believe that the facile and rapid method of synthesis of MFIR NLO SbI_3_·3S_8_ crystal might be a potential candidate for the future of optoelectronic devices.

## 2. Materials and Methods

### 2.1. Materials

Antimony (Sb, 99.95%) was procured from Sigma–Aldrich. Sublimated sulfur (S), iodine (I), and dichloromethane (CH_2_Cl_2_) were bought from POCH S.A. (Gliwice, Poland). All the materials were used for experimental purposes without further purification. Ethanol and deionized water were engaged in the separation and purification process.

### 2.2. Synthesis of SbI_3_·3S_8_ Crystal

**Figure 2:**
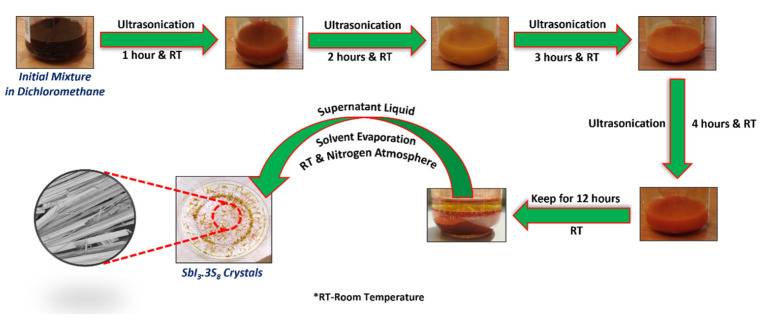
Digital images of each step of the synthesis of SbI_3_·3S_8_ crystal.

In a typical method, predetermined weights of antimony (Sb), sulfur (S), and iodine (I) were taken in 20 mL of dichloromethane (CH_2_Cl_2_) containing closed vessels and sonicated for 4 h in an ultrasonication bath. After sonication, the solution mixture was kept for 12 h for the settlement of solid particles. Later, the supernatant liquid was carefully poured into a Petri dish from the above portion of the mixture, and the Petri dish containing the supernatant liquid was kept in a nitrogen atmosphere for gradual evaporation of the solvent. As soon as we kept the Petri dish in the nitrogen atmosphere, the solvent instantly evaporated from the Petri dish with the formation of a yellow crystal, and the process of crystallization was completed within 2 h (at that same time evaporation of dichloromethane was completed). After the complete evaporation of the solvent, the grown crystals were collected from the Petri dish and kept in a closed chamber for further investigation and characterization purposes. The yield of the products was around 0.150 gm. The steps of SbI_3_·3S_8_ crystal formation starting from raw materials are schematically presented in Figure 2.

### 2.3. Characterization Techniques

Optical microscope imaging of the grown SbI_3_·3S_8_ crystals was performed with Stemi 2000-C Stereo Microscope (Zeiss, Germany) equipped with an Olympus DP25 camera (Olympus, Japan). Data acquisition from the microscope was performed using the Olympus Stream Basic 1.9 program. Powder X-ray diffraction (XRD) measurements of the grown SbI_3_·3S_8_ crystal were performed using a Panalytical Empyrean diffractometer (Malvern Instruments, Malvern, UK) with a Cu anode (with a wavelength of 1.54056 Å) working at an electric current of 30 mA and a voltage of 40 kV and equipped with an ultrafast solid-state hybrid detector (Malvern Instruments, Malvern, UK). The X-ray diffraction measurements were performed in an angular range of 2θ from 5° to 90° with a 0.02° step in Bragg–Brentano geometry (θ–θ scan technique), and the time count was 1000 s for each point at room temperature T ≈ 300 K. The morphology and elemental composition of the synthesized SbI_3_·3S_8_ crystal were examined by scanning electron microscopy (SEM) and energy-dispersive X-ray spectroscopy (EDX). These analyses were executed through the Phenom Pro X (Phenom World) microscope integrated with the EDX spectrometer. The peaks in the EDS spectrum were investigated by the ProSuite Element Identification (Phenom-World) computer program. The optical transmittance spectra of the SbI_3_·3S_8_ single crystals were measured at room temperature using the Flame spectrophotometer equipped with waveguide cables and the deuterium–halogen light source from Ocean Optics. Schematic diagram and a photo of the measuring station used to obtain the generation of the second harmonic of light are presented in Section 5. Second Harmonic Generation of Light in the SbI_3_·3S_8_. The source of 1064 nm radiation was an Nd:YAG/Cr:YAG microlaser pumped by a LIMO25-F100-LD808 laser diode with fiber optic output. The microlaser generated radiation pulses with a duration of 3 ns and energy of approx. 14 µJ. The repetition frequency varied linearly as a function of the pumping power. Radiation with a wavelength of 1064 nm was directed to the partitioning plate with a transmission of 94%. The reflected part of the radiation was recorded with an InGaAs ET-3000 photodiode to measure the temporal characteristics of the pulses. The output from the photodiode was observed on an Agilent Technologies MSO7104A oscilloscope. To eliminate the pumping radiation, the laser beam was directed to a dichroic mirror reflecting the radiation with a wavelength of 1064 nm.

## 3. Results

**Figure 3:**
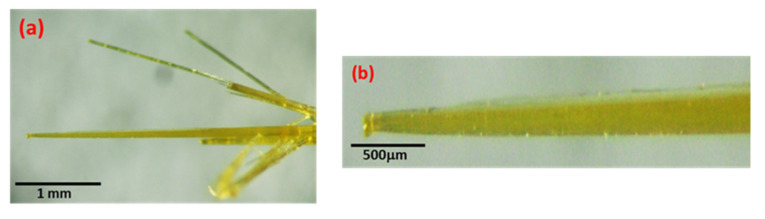
Optical microscopic images of grown (**a**) branches and (**b**) single SbI_3_·3S_8_ crystals.

The optical microscopic (OM) images of the synthesized bright yellow branched SbI_3_·3S_8_ crystal as well, and single SbI_3_·3S_8_ crystals are presented in Figure 3a,b, respectively. The images clearly show that the length of the crystals varies in the range of 1–5 mm, while the diameter is in the range of 40–100 µm. When the single crystal is observed at a higher magnification by the optical microscope, a spherical drop is noticed at the end of the crystal, suggesting the termination of the crystallization process. Most likely, these droplets are formed by analogy to the mechanism of dew formation, e.g., on grass. Particles of crystal-forming substances settle on it. They form a thin layer that transforms into a droplet. For this to happen, the air temperature must drop below the dew point.

**Figure 4:**
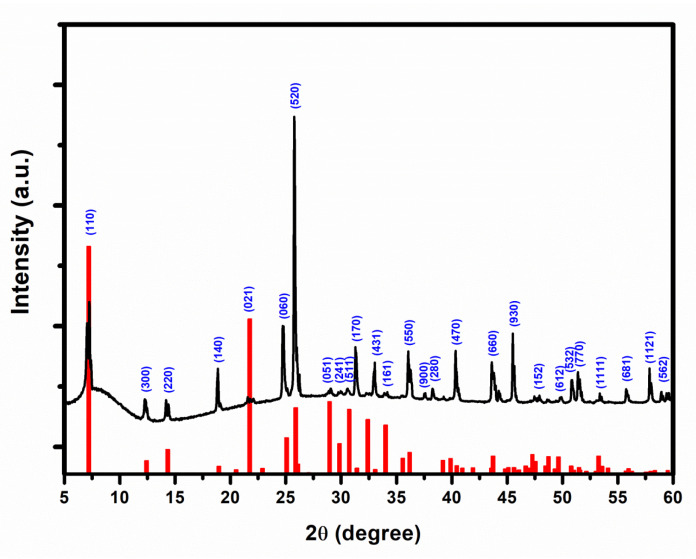
X-ray diffraction (XRD) pattern of prepared SbI_3_·3S_8_ crystal (The peaks are marked with crystal planes and below provide the JCPDS (PDF No. 71-2024) of SbI_3_·3S_8_ crystal).

To reveal the crystallinity, purity, and crystallite size of the grown crystals, we performed an X-ray diffraction experiment, and the results are demonstrated in Figure 4. The sharp and distinct peaks indicate a well-defined crystallized structure of synthesized SbI_3_·3S_8_ crystal. The diffraction peaks are well-matched with the hexagonal R3m structure (PDF No. 71-2024), and the absence of other peaks indicates a high purity of the synthesized crystal [29]. The comparison between the crystal planes with diffraction angles (2θ) of the XRD experiment and the values obtained from standard 2θ of JCPDS (PDF No. 71-2024) of SbI_3_·3S_8_ crystal are summarized in Table 1. The table clearly shows that the theoretical and experimental values are similar, with minor deviations indicating a high purity of the crystals. To determine the interplanar spacing and crystallite size using Bragg’s law and the Debye–Scherrer equation, respectively, we use an exposed (110) crystal plane, and the measured value of the interplanar spacing and crystallite size are 1.22 nm and 282.17 nm, respectively.

**Figure 5:**
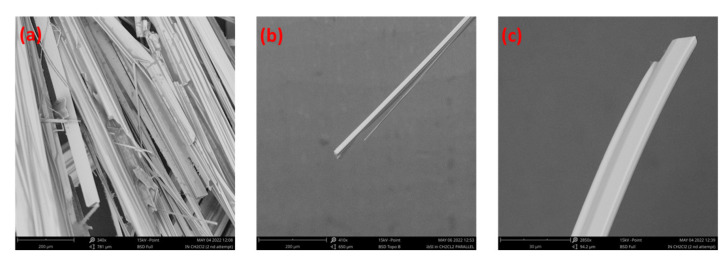
(**a**) SEM image of the branch of SbI_3_·3S_8_ crystal. (**b**,**c**) SEM images of a single SbI_3_·3S_8_ crystal after rotating the silicon wafer at a specific angle.

To further study the morphology and internal composition of the SbI_3_·3S_8_ grown crystal, we examined the crystal using SEM, and the corresponding results are presented in Figure 5. Figure 5a shows groups of synthesized SbI_3_·3S_8_ crystals, and the images also show that the materials have slab-like structures with high purity and good crystallinity. The crystals are oriented in a particular direction. To achieve better morphology of the synthesized crystals, we examined the crystals under SEM after placing the crystal in a perpendicular direction to that of the silicon wafer, and the results are demonstrated in Figure 5b,c.

**Figure 6:**
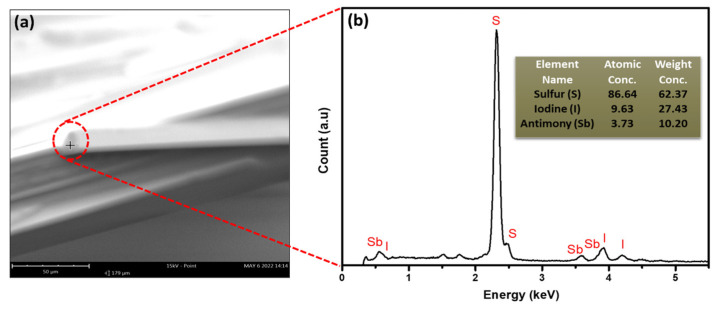
(**a**) SEM image of SbI_3_·3S_8_ crystal and (**b**) energy-dispersive X-ray spectroscopy (EDS) of SbI_3_·3S_8_ crystal (inset of the EDS spectrum demonstrates the atomic concentration and weight concentration of each element present in the crystal).

**Figure 7:**
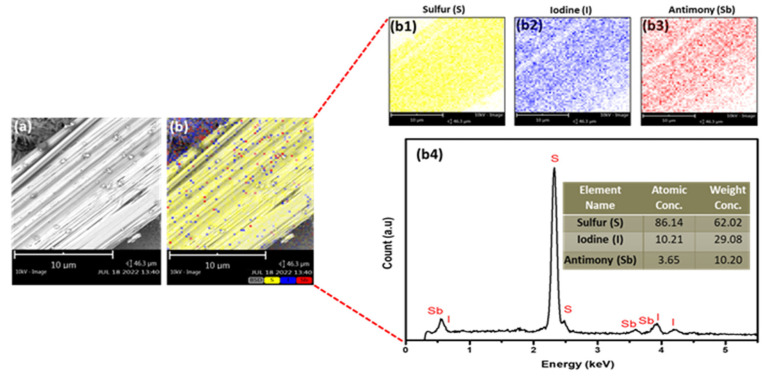
(**a**) SEM image and (**b**) EDS mapping and elemental analysis including (**b1**) sulfur (S), (**b2**) iodine (I), (**b3**) antimony (Sb), and (**b4**) EDS spectrum (inset shows the atomic concentration and weight concentration of each element present in the crystal) of the SbI_3_·3S_8_ crystal.

The figures show the well-aligned, uniform, and hexagonal morphology of SbI_3_·3S_8_ crystal without any impurity and a better outlook of the single crystal. To quantify the elemental composition of the as-grown SbI_3_·3S_8_ crystal, we performed an energy-dispersive X-ray spectroscopy (EDX) study during SEM analysis, and the results of this study are summarized in Figure 6. The EDX spectra show that the atomic ratio of S, I, and Sb in the synthesized SbI_3_·3S_8_ crystal is very close to 24:3:1, and the stoichiometric ratio of the elements in the crystal have good similarity with the theoretical composition of SbI_3_·3S_8_ crystal. The distribution of elements throughout the crystal was examined using elemental mapping, and the corresponding outcomes are demonstrated in Figure 7. The elemental mapping reports a uniform distribution of S, I, and Sb in the whole crystal. The results of the SEM study confirm the successful synthesis of SbI_3_·3S_8_ crystal.

**Figure 8:**
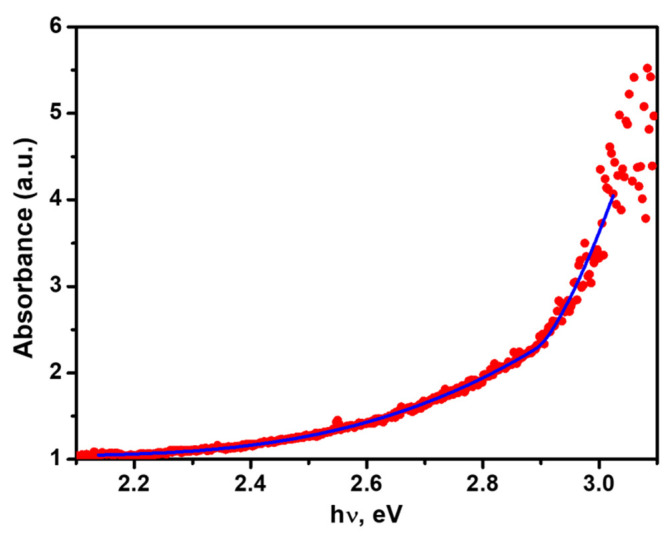
Absorbance spectrum of SbI_3_·3S_8_ single crystal measured at room temperature. The solid curve represents the least square fitted theoretical dependences for the sum of constant absorption term, direct and indirect forbidden absorptions, and Urbach ruled absorption (description and values of the fitted parameters are given in the text).

Figure 8 presents the absorbance spectra of one of the SbI_3_·3S_8_ single crystals calculated from optical transmittance. Applying the method of simultaneous fitting of many absorption mechanisms to the spectral dependence of absorbance [25], these data were used to determine the optical energy gap of the SbI_3_·3S_8_ single crystal. The red curve from Figure 8 presents the best-fitted theoretical dependence of the investigated SbI_3_·3S_8_ single crystal appropriate for the sum of indirect forbidden absorption without excitons or phonon statistics (α_1_), direct forbidden absorption without excitons (α_2_), Urbach absorption (α_3_), and constant absorption term (α_4_) [30]:

(1) $$\alpha_{1}=A_{60}{\left(h\nu -E_{gIf}\right)}^{3}\;for\;h\nu >E_{gIf}$$

(2) $$\;\alpha_{2}=\frac{A_{3}}{h\nu }{\left(h\nu -E_{gDf}\right)}^{3/2}\;for\;h\nu >E_{gDf}$$

(3) $$\;\alpha_{3}=A_{U}\exp\left(\frac{h\nu }{E_{U}\;}\right)=A\exp\left(\frac{B\left(h\nu -C\right)}{k_{B}T\;}\right)$$

(4) $$\alpha_{4}=A_{0}$$

where E_gIf_ represents the indirect forbidden energy gap; E_gDf_ is the direct forbidden energy gap; E_U_ is the Urbach energy; and A_60_, A_3_, and A_U_ are constant parameters. The constant absorption term A_0_ is an attenuation coefficient that is considered the sum of the scattering and absorption independent of photon energy (hν) near the absorption edge. The values of the fitted parameters are: E_gDf_ = 2.893 eV, E_gIf_ = 1.986 eV, E_U_ = 38.8 meV, A_3_ = 73.4 eV^−1/2^, A_60_ = 1.665 eV^−3^, A_U_ = 1.609 × 10^−3^, and A_0_ = 1.045.

## 4. Plausible Mechanism of Formation of SbI_3_·3S_8_ Crystal in CH_2_Cl_2_

The possible mechanism of SbI_3_·3S_8_ crystal formation from elemental Sb, S, and I in dichloromethane as a solvent medium under ultrasonic irradiation can be described as follows:

(i) First, due to the high reactivity tendency of iodine (I) and antimony (Sb), they easily react with each other in dichloromethane (CH_2_Cl_2_) to form antimony tri-iodide (SbI_3_).

2Sb + 3I_2_
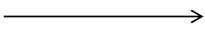
2SbI_3_(5)

(ii) At the same time, during ultrasonication, CH_2_Cl_2_ will break down into various reactive radical species [31]. The steps of radical formation are given below:

CH_2_Cl_2_
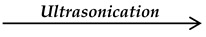
H· + ·CHCl_2_(6)

H· + CH_2_Cl_2_
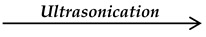
H_2_ ↑ + ·CHCl_2_(7)

(iii) In the next step, the highly reactive radicals react with iodine molecules of SbI_3_ molecules through the formation of an adduct compound. The intermediate compounds are very unstable and tend to react further.

SbI_3_+3·CHCl_2_
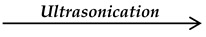
SbI_3_·3CHCl_2_(8)

(iv) On the other side, when ultrasonic waves passed through a liquid medium, they stretched and compressed the molecules in which they were transmitted. During the stretching period, negative pressure is created on the liquid medium and at a specific pressure; they produced microbubbles when the pressure surpassed the intermolecular binding forces of liquid molecules. In the next cycles of stretching and compression, these microbubbles grew continuously and decomposed near the surface of the elemental sulfur. During the decomposition of these bubbles, they generated high temperature and pressure, which is responsible for the cleavage of elemental sulfur into small S_8_ molecules [32].

Elemental sulfur
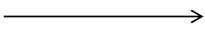
nS_8_
(9)

(v) These highly reactive small S_8_ molecules can gradually diffuse near the unstable SbI_3_·3CHCl_2_ adduct compound, and they reacted through a substitution reaction to form SbI_3_·3S_8_ nuclei.

3S_8_ + SbI_3_·3CHCl_2_
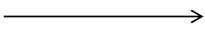
SbI_3_·3S_8_(10)

(vi) The formed nuclei are unstable in the solvent medium, and they always have a propensity to grow as SbI_3_·3S_8_ crystals along a specific axis by sharing lone pair electrons of Sb molecules. Room temperature evaporation of the solvent (CH_2_Cl_2_), as well as the attraction of lone pairs, helps in the formation of a one-dimensional crystal structure by pushing the nuclei to each other. Finally, we obtained the grown crystal, which is one-dimensional and highly anisotropic in nature [27,33].

**Figure 9:**
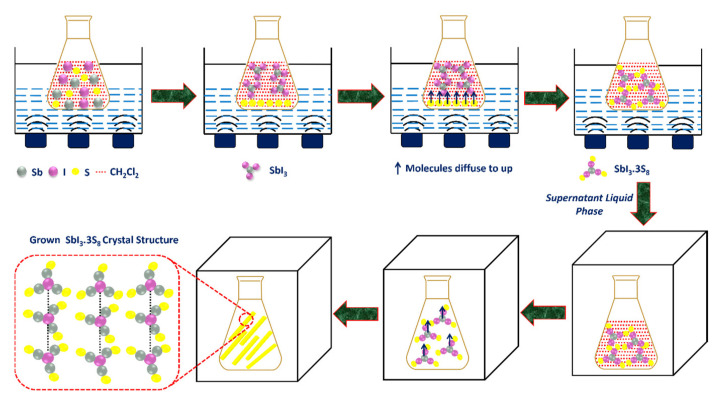
Schematic diagram of the crystal the mechanism of grown SbI_3_·3S_8_ crystal starting from raw materials (antimony (Sb), iodine (I), and sulfur (S)) in dichloromethane (CH_2_Cl_2_) as a solvent medium.

The steps of the plausible formation mechanism of SbI_3_·3S_8_ crystals are also schematically presented in Figure 9.

## 5. Second Harmonic Generation of Light in the SbI_3_·3S_8_

**Figure 10:**
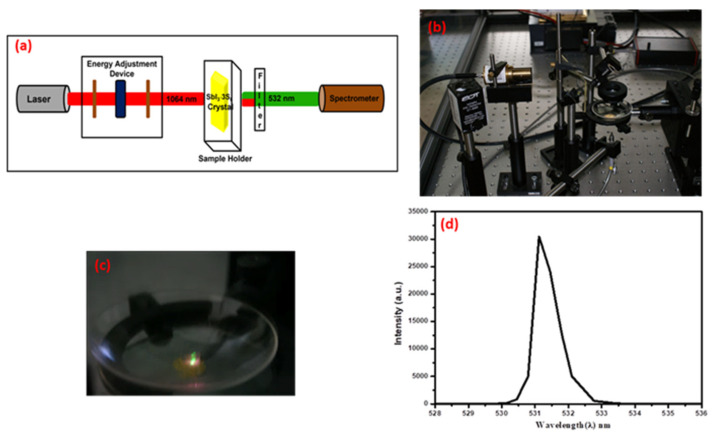
(**a**) Schematic diagram of the experimental set-up of SHG. (**b**) Digital image of the test stand for SHG generation in SbI_3_·3S_8_ crystals, (**c**) a shining piece of the crystal SbI_3_·3S_8_ during the generation of the second harmonic of light, and (**d**) spectral characteristics of the second harmonic of light generation for the repetition frequency of 0.85 kHz.

A radiation level of 1064 nm was focused on the test material using a lens with a focal length of 35 mm. The examined crushed pieces of SbI_3_·3S_8_ crystals were placed in a glass container (with a transmitting radiation of 1064 nm and 532 nm) on a micrometric table, enabling movement on three axes. Behind the tested sample, there were band-stop and band-rejection Notch filters (NF1064-44—Ø25 mm Notch Filter, CWL = 1064 nm, FWHM = 44 nm, ThorLabs) that cut off 1064 nm radiation while maintaining high transmission at 532 nm radiation. The radiation of 532 nm was carried by a fiber with a core diameter of 230 µm to a computer-controlled Avantes AvaSpec-3648 spectrometer. The schematic diagram with pathways of lights and a digital image of the experimental set up are presented in Figure 10a,b. The photo in Figure 10c shows a green glowing signal SbI_3_·3S_8_ crystal. The generation spectrum is shown in Figure 10d. The spectrum shows that the intensity of the peak is much higher than previously published results [20,28], which accounts for the high purity of the synthesized SbI_3_·3S_8_ crystals.

## 6. Conclusions

In summary, an inorganic adduct of MFIR SbI_3_·3S_8_ crystals was synthesized by a facile liquid phase reaction through the consecutive combined action of the ultrasonic irradiation and evaporation method for the first time from the individual elements (Sb, I, and S). The formation of SbI_3_·3S_8_ crystals was confirmed by various well-known characterized techniques, and based on this, a probable mechanism of the crystals is represented. The study of the NLO properties suggests SbI_3_·3S_8_ crystals exhibit a second-harmonic generation response, which is presented as a potential candidate for NLO crystals in the MFIR region. Virtually, this work broadly scrutinized an MFIR NLO crystal, and their exceedingly encouraging NLO properties and the need for consideration of all-inorganic adduct-type NLO properties project a critical inquiry about subgroups for investigation of novel NLO crystals. It is anticipated that this work can inspire more analysis of seldom-touched inorganic adducts. Moreover, its basic chemical composition, simple synthesis, and high productivity make it one of the foremost promising MFIR NLO crystals for futuristic optoelectronic devices. The results presented, and in particular the new fast and cheap method of SbI_3_·3S_8_ production, may constitute the basis for more detailed research on the SHG phenomenon observed in this material.

## Figures and Tables

**Table 1:** Comparison of a diffraction angle (2Ɵ) from the results obtained from the XRD experiment with standard values for the SbI_3_·3S_8_ crystal.

SL. No.	Crystal Planes			Observed 2Ɵ from XRD Experiment (Degree)	Standard 2Ɵ from JCPDS (PDF No. 71-2024) (Degree)
	h	k	l		
**1.**	1	1	0	7.282	7.118
**2.**	3	0	0	12.350	12.345
**3.**	2	2	0	14.344	14.264
**4.**	1	4	0	18.909	18.907
**5.**	0	2	1	21.890	21.690
**6.**	0	6	0	24.782	24.836
**7.**	5	2	0	25.779	25.868
**8.**	0	5	1	28.962	28.962
**9.**	2	4	1	29.947	29.826
**10.**	5	1	1	30.644	30.702
**11.**	1	7	0	31.340	31.399
**12.**	4	3	1	32.650	32.390
**13.**	1	6	1	34.027	34.004
**14.**	5	5	0	36.121	36.166
**15.**	9	0	0	37.610	37.637
**16.**	2	8	0	38.307	38.354
**17.**	4	7	0	40.289	40.441
**18.**	6	6	0	43.671	43.761
**19.**	9	3	0	45.466	45.620
**20.**	1	5	2	47.435	47.292
**21.**	6	1	2	49.586	49.652
**22.**	5	3	2	50.835	50.801
**23.**	7	7	0	51.523	51.513
**24.**	11	1	1	53.321	53.289
**25.**	6	8	1	55.808	55.997
**26.**	11	2	1	57.698	57.580
**27.**	5	6	2	58.786	58.392

## Data Availability

All data presented in this study are included in the published article.

## References

[1] Kang L., Liang F., Jiang X., Lin Z., Chen C. (2019). First-principles design and simulations promote the development of nonlinear optical crystals. Acc. Chem. Res..

[2] Kang L., Lin Z. (2022). Deep-ultraviolet nonlinear optical crystals: Concept development and materials discovery. Light Sci. Appl..

[3] Mutailipu M., Poeppelmeier K.R., Pan S. (2020). Borates: A rich source for optical materials. Chem. Rev..

[4] Xu B., Li Z., Wang K., Zhang J., Liang L., Li L., Ren Y., Liu Y., Liu M., Xue D. (2021). Growth and Optical Properties of the Whole System of Li (Mn_1−x_, Ni_x_) PO_4_ (0 ≤ x ≤ 0.5) Single Crystals. Materials.

[5] Shen Y., Zhao S., Luo J. (2018). The role of cations in second-order nonlinear optical materials based on π-conjugated [BO_3_]^3−^ groups. Coord. Chem. Rev..

[6] Guo S.-P., Chi Y., Guo G.-C. (2017). Recent achievements on middle and far-infrared second-order nonlinear optical materials. Coord. Chem. Rev..

[7] Zhao J., Wu J., Chen X., Zeng R. (2022). Effect of Temperature on Ultrasonic Nonlinear Parameters of Carbonated Concrete. Materials.

[8] Samoc A., Samoc M., Luther-Davies B., Kelly J., Krausz E., Willis A. (2004). New second-order nonlinear octupolar materials. Mol. Cryst. Liq. Cryst..

[9] Lin H., Zheng Y.J., Hu X.N., Chen H., Yu J.S., Wu L.M. (2017). Non-centrosymmetric Selenides AZn_4_In_5_Se_12_ (A = Rb, Cs): Synthesis, Characterization and Nonlinear Optical Properties. Chem. Asia. J..

[10] Liu K., Kang Y., Tao H., Zhang X., Xu Y. (2022). Effect of Se on Structure and Electrical Properties of Ge-As-Te Glass. Materials.

[11] Chung I., Kanatzidis M.G. (2014). Metal chalcogenides: A rich source of nonlinear optical materials. Chem. Mater..

[12] Yu H., Koocher N.Z., Rondinelli J.M., Halasyamani P.S. (2018). Pb_2_BO_3_I: A Borate Iodide with the Largest Second-Harmonic Generation (SHG) Response in the KBe_2_BO_3_F_2_ (KBBF) Family of Nonlinear Optical (NLO) Materials. Angew. Chem. Int. Ed..

[13] Pan Y., Guo S.-P., Liu B.-W., Xue H.-G., Guo G.-C. (2018). Second-order nonlinear optical crystals with mixed anions. Coord. Chem. Rev..

[14] Sun Z.-D., Chi Y., Xue H.-G., Guo S.-P. (2017). A Series of Pentanary Inorganic Supramolecular Sulfides (A_3_X) [MB_12_(MS_4_)_3_] (A = K, Cs; X = Cl, Br, I; M = Ga, In, Gd) Featuring B_12_S_12_ Clusters. Inorg. Chem. Front..

[15] Scherbak S.A., Kaasik V.P., Zhurikhina V.V., Lipovskii A.A. (2022). Poling of Glasses Using Resistive Barrier Discharge Plasma. Materials.

[16] Sekar A., Muthurakku U.R., Sivaperuman K. (2021). An Overview on Recent Trends in Deep-Ultraviolet (DUV) and Ultraviolet (UV) Nonlinear Optical Crystals. ChemistrySelect.

[17] Mutailipu M., Yang Z., Pan S. (2021). Toward the enhancement of critical performance for deep-ultraviolet frequency-doubling crystals utilizing covalent tetrahedra. Acc. Mater. Res..

[18] Janarthanan S., Samuel R.S., Selvakumar S., Rajan Y., Jayaraman D., Pandi S. (2011). Growth and characterization of organic NLO crystal: β-naphthol. J. Mater. Sci. Technol..

[19] Almuqrin A.H., Gangareddy J., Hivrekar M.M., Pramod A., Sayyed M., Keshavamurthy K., Fatima N., Jadhav K. (2022). Nonlinear Optical Limiting and Radiation Shielding Characteristics of Sm_2_O_3_ Doped Cadmium Sodium Lithium Borate Glasses. Materials.

[20] Lu Z.-T., Sun Z.-D., Chi Y., Xue H.-G., Guo S.-P. (2019). Balanced second-order nonlinear optical properties of adducts CHI_3_·(S_8_)_3_ and AsI_3_·(S_8_)_3_: A systematic survey. Inorg. Chem..

[21] Shui Q.R., Fu R.B., Zhou Z.Q., Ma Z.J., Tang H.X., Wu X.T. (2022). A Lead Mixed Halide with Three Different Coordinated Anions and Strong Second-Harmonic Generation Response. Chem. Euro. J..

[22] Chen C., Wu Y., Li R. (1989). The anionic group theory of the non-linear optical effect and its applications in the development of new high-quality NLO crystals in the borate series. Int. Rev. Phys. Chem..

[23] Yan M., Xue H.-G., Guo S.-P. (2020). Recent achievements in lone-pair cation-based infrared second-order nonlinear optical materials. Cryst. Growth Des..

[24] Xiao J.-R., Yang S.-H., Feng F., Xue H.-G., Guo S.-P. (2017). A review of the structural chemistry and physical properties of metal chalcogenide halides. Coord. Chem. Rev..

[25] Nowak M., Kotyczka-Morańska M., Szperlich P., Jesionek M., Kępińska M., Stróż D., Kusz J., Szala J., Moskal G., Rzychoń T. (2010). Using of sonochemically prepared components for vapor phase growing of SbI_3_·3S_8_. Ultrason. Sonochemistry.

[26] Fernando W. (1981). Single crystal Raman spectra of SbI_3_·3S_8_; CHI_3_·3S_8_ and AsI_3_·3S_8_. J. Inorg. Nucl. Chem..

[27] Bjorvatten T., Hassel O., Lindheim A. (1963). Crystal structure of addition compound SBI_3_/3S_8_. Acta Chem. Scand..

[28] Guo S.-P., Sun Z.-D., Chi Y., Xue H.-G. (2018). Adduct-type IR nonlinear-optical crystal SbI_3_·(S_8_)_3_ with a large second-harmonic generation and a high laser-induced damage threshold. Inorg. Chem..

[29] Feng X., Sun Z., Pei K., Han W., Wang F., Luo P., Su J., Zuo N., Liu G., Li H. (2020). 2D inorganic bimolecular crystals with strong in-plane anisotropy for second-order nonlinear optics. Adv. Mater..

[30] Kotyczka-Morańska M., Nowak M., Kępińska M., Szperlich P., Szala J. (2008). Własności optyczne monokryształów SbI_3_·3S_8_ otrzymanych z fazy gazowej. Inż. Mater..

[31] Lim M., Son Y., Khim J. (2011). Frequency effects on the sonochemical degradation of chlorinated compounds. Ultrason. Sonochem..

[32] Warren B., Burwell J. (1935). The structure of rhombic sulphur. J. Chem. Phys..

[33] Gedanken A. (2004). Using sonochemistry for the fabrication of nanomaterials. Ultrason. Sonochem..

